# Chemistries Moonshot: An Entirely Recyclable Car

**DOI:** 10.1021/acscentsci.5c00589

**Published:** 2025-07-02

**Authors:** Robin Schoemaker, Chunning Sun, Davide Chiarugi, Theodore Tyrikos-Ergas, Peter H. Seeberger

**Affiliations:** † Biomolecular Systems Department, 28321 Max-Planck-Institute of Colloids and Interfaces, Potsdam 14476, Germany; ‡ Center for the Transformation of Chemistry (CTC), Leuna 06237, Germany; § Freie Universität Berlin, Institute of Chemistry and Biochemistry, Berlin 14195, Germany

## Abstract

Automobiles depend on fossil resources – both to create the device and to power it. The automotive industry has decreased this dependency on fossil fuels by developing more fuel-efficient combustion engines, lightweight designs, and biofuels. The rise of battery electric vehicles (BEVs) offers the chance to reduce the fossil footprint by avoiding fuel combustion and exhaust emission. Disruptive approaches toward a truly sustainable car are far from being market-ready. To reach a completely sustainable car, the automotive industry must address the carbon footprint of material production, which is based in the chemical sector. The automotive and chemical industries have to adopt closed-loop thinking, utilize renewable resources for biodegradables, as well as develop novel materials and designs for efficient recycling. Disruptive approaches can arise from predictive models that can accelerate chemical research and enable the discovery of sustainable materials with desirable recycling properties. Integrating generative artificial intelligence (AI) with high-throughput experimental validation will shorten material development cycles and advance the transition to more sustainable products. Moving toward a fully recyclable car is aligning research and development efforts from the chemical sector to the automotive industry and beyond, presenting a giant leap toward a circular economy.

## Graphical abstract

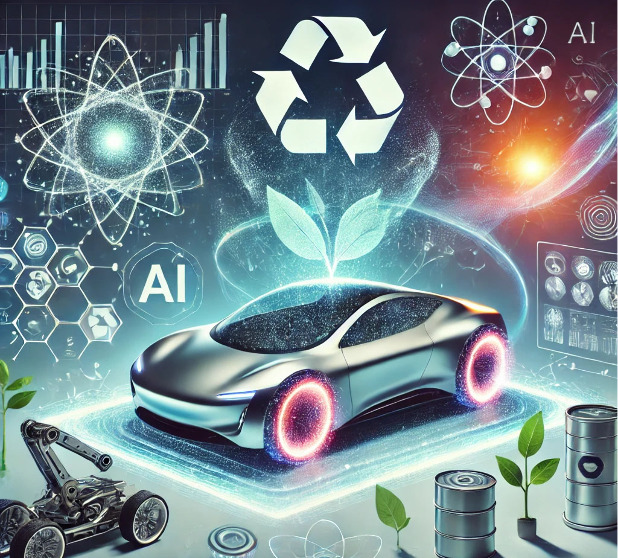

## Introduction

Fossil fuels have historically provided energy for vehicles and raw materials for plastics, coatings, and other components. These resources are associated with well-documented environmental drawbacks, including high greenhouse gas emissions and a linear lifecycle that generates substantial waste.^1^

Fuel combustion and exhaust emissions account for 65–80%^1^ of the total lifecycle emissions (Figure 1), for internal combustion engine vehicles (ICEVs). Phasing out ICEVs in favor of electric powertrains can substantially reduce use-phase emissions. At the same time, the share of material production, will surge from currently 18–22% of total emissions from ICEVs, to 60%, to dominate lifecycle emissions (Figure 1). Material emissions for BEV can be twice as much as for ICEV largely due to energy intensive battery production.^1^ As a result, the balance of a car’s lifetime emissions moves from the use phase to production, indicating that phasing out ICEVs alone will not suffice to drastically cut carbon emissions. The automotive sector must address the carbon footprint of material production, an energy- and emission-intensive process that places new demands on industry-wide defossilization efforts.^2–4^ Materials derived from naturally replenishing resources present a promising alternative together with a complete recyclability at a car’s end of life.^5^ Creating such a fully recyclable car will be a single, unifying project for material chemistry. Similar to the Apollo Program, the Human Genome Project, and CERN (European Organization for Nuclear Research) that each made significant contributions to their respective fields of research. These “moonshot” programs have had a profound impact on aligning scientists’ efforts and advancing their respective as well as neighboring fields.^6^ Chemistry is a foundational scientific discipline with a broad and multifaceted impact, influencing various sectors of the economy and numerous fields of science and aided the aforementioned moonshot programs.^7–14^ While chemistry will be a major part of this grand challenge, it goes beyond that due to its inherent interdisciplinary nature. In addition to new materials, a new way of thinking about these materials is necessary. This new way of thinking should start with research including assumptions on life cycles. Therefore, material chemists need input from systems engineering, industrial ecology and sustainability science, especially when thinking of new developments in the field of data sciences and AI.^15^ The goal is to holistically develop materials from cradle to cradle instead of synthesizing them to perform before discarding them.^16^ Materials chemistry is inviting all scientific fields in question from automotive, environmental or systems engineering to data science, recycling and waste management on a journey to the moon.

**Figure 1:**
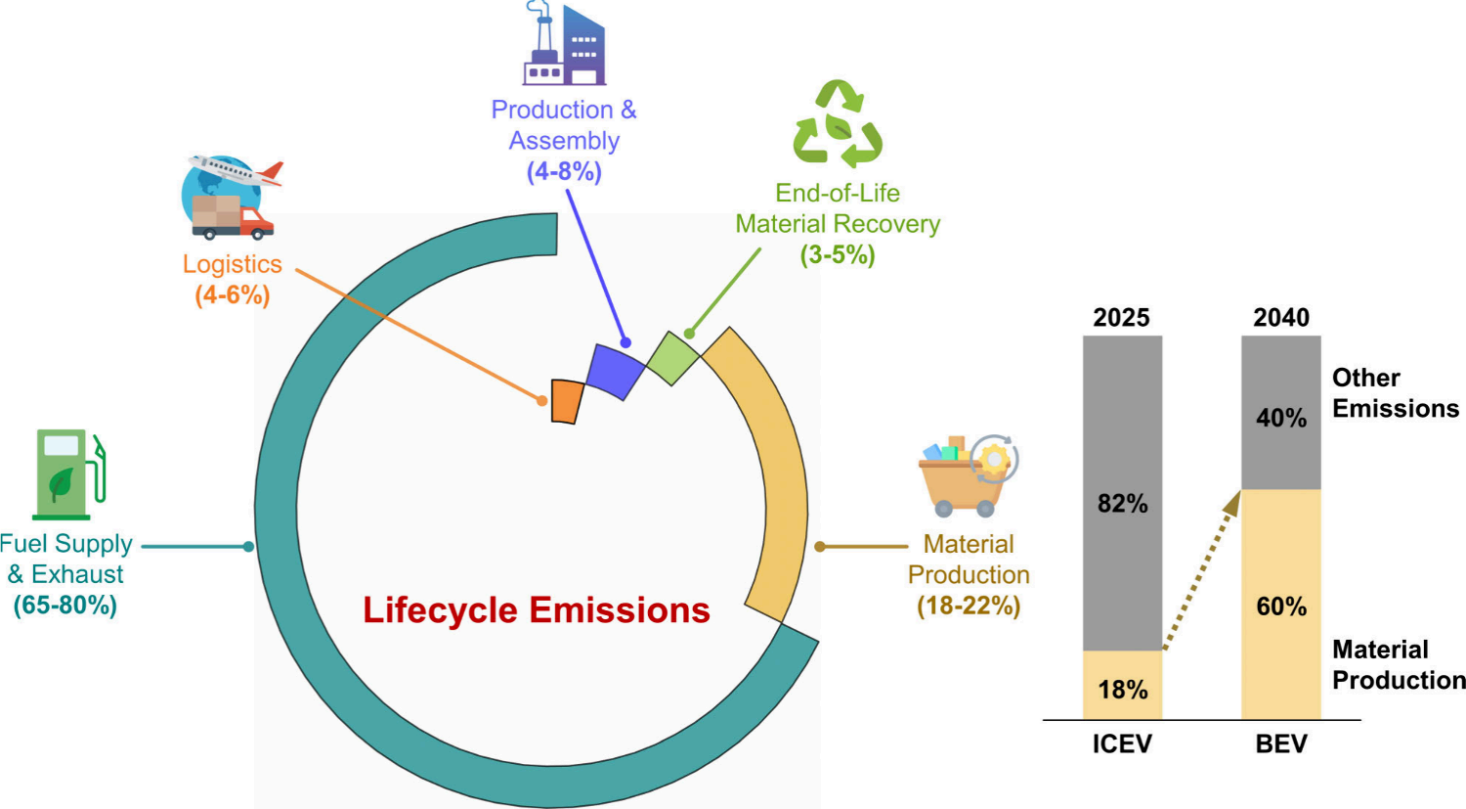
Share of total lifecycle emissions of internal combustion engine vehicles (left) and expected change in emissions of material production (right).^1^

This journey has the potential to initiate significant efforts in both industry and academia, science and engineering.^17–20^ In order to develop a recyclable car, one must first understand the current materials used in the automotive industry, practices in end-of-life vehicle (ELV) treatment and what challenges lie on this way transforming life cycles from cradle to grave to cradle to cradle.^21^ We will discuss the most significant challenges and potential solutions to achieving a recyclable automobile focusing on recent developments toward a data-driven chemistry.

## The Recyclable Car: What Is Recycling?

Recycling is a vague, ambiguous and sometimes misleading term. Waste incineration is sometimes called “recycling to energy”. Thinking of recycling as a loop of matter this is actually not a recycling approach, as all matter resulting from incineration is either landfilled and/or released into the atmosphere and leaves the loop. Other materials are processed and reused in other, often less demanding applications, before they reach the end of their useful life. This is also called recycling or more precisely downcycling, as it merely postpones the inevitable issue. For us, recycling should mean cradle-to-cradle design^22^ since by truly closing the loop, a continuous, sustainable and truly circular economy is within reach.^23^ At the same time, there are inevitably expendable parts and materials that leave the life cycle. Developing a recyclable car means to create materials that are either biodegradable or recyclable, thereby integrating into the technical or biological cycle (Figure 2). Biodegradable materials are essential for components such as tires or brake pads that enter the environment through vehicle use. These materials have to contribute to renewable biomass after biodegradation by forming biological nutrients, thus closing the cycle. Another alternative is to harvest these biological nutrients and use them directly in production, e.g. in CO_2_ to X processes.^24,25^ The technical cycle involves collecting and disassembling end-of-life vehicles. After separating car parts and materials, they can be reused directly or converted into valuable resources for producing new cars.

**Figure 2:**
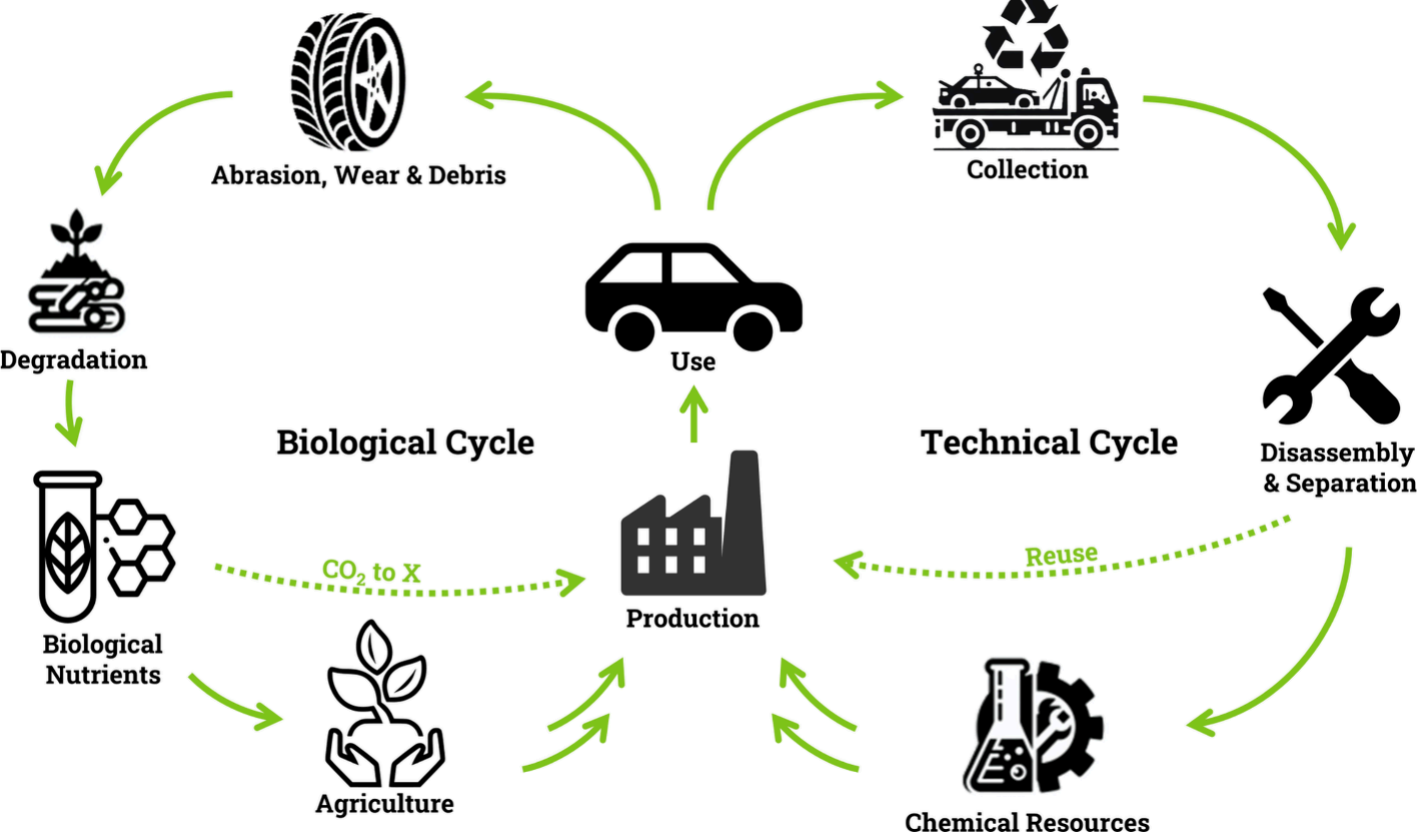
An automotive’s life adhering to the cradle-to-cradle principle.^22^

Recycling is a vague, ambiguous and sometimes misleading term.

## Current Status and Challenges in ELV Recycling

ELV recycling is a complex mechanical procedure commencing with decontamination to eliminate all hazardous substances followed by scrapping. Single components may enter the secondary market directly or after refurbishment. Parts deemed unsuitable for that purpose are shredded into fist-sized fragments, that are fractionated into ferrous, nonferrous, and nonmetallic materials. The metallic fractions are sent to metallurgical recycling streams, while the automotive shredder residue (ASR) fraction is rarely recycled and often incinerated or landfilled.^26^ Some car parts and materials are already recycled, while others are still treated as waste (Table 1).

**Table 1:** ELV Parts and Components with Recyclability Estimation and Posing Challenges

ELV parts/materials	recyclability (−, o, + )	status quo and posing challenges
Fuel	–	Incinerated and nonrecyclable by default.
Lubricants and oils	o	Some rerefining possible, leaving some residual waste. Renewably resourced lubricants are a solution.^27,28^
Tires	o	Generally, ground and used as filler in construction industry or fuel in cement kilns. Tire pyrolysis produces pyrolysis gas and oil as well as carbon black for reuse in tires. Tire devulcanization is currently explored.^29–38^
Glass	o	Packing glass can be recycled indefinitely. ELV windshields are layered with polyvinylbutyrate or other plastics, prevent shards and recycling. ELV glass collection is insufficient and limits recycling.^39–45^
Batteries	+ for Pb batteries	Lead-acid starter batteries are collected and recycled with rates reaching 100% in developed countries.^46^
	o/+ for Li-ion and nickel–metal hydride (NiMH) batteries	Rising numbers of BEV, Li-ion, and NiMH battery recycling are gaining momentum. Infrastructure established to prevent Li, Co, Ni, Mn, Al, Cu, and graphite shortages.^47^
Ferrous metals	+	Recycled effectively. Steel industry is relying on ELV scrap.^48^
Nonferrous metals	o/+	Aluminum and copper are recycled due to economic benefits compared to Al and Cu from primary resources. Alloy formation can lower quality; solutions for this are explored.^49–51^
Aluminum	+	
Copper		
Platinum group metals (PGMs)	+	Pd, Pt, and Rh used for exhaust purification. Spent automotive catalysts (SACs) are easily collected. High prices for PGMs make recycling economically favorable. Multiple companies recycle PGMs from SAC and other secondary resources like electronic waste and spent industry catalysts. Shift from ICEV to BEV might lower demand for Pd, Pt, and Rh, while demand for Ir and Ru, used in electronics, might rise.^52–56^
Plastics	+ larger thermoplastics—other plastic parts → ASR	Larger plastic parts might be disassembled and collected for mechanical recycling, working very well for pure streams of thermoplastics like polypropylene (PP).^57,58^ Other plastics are collected in the ASR that is usually incinerated or pyrolyzed due to its relatively high carbon content >40%.^59–65^

Metals account for the largest share of a car by weight, for ICEB and BEV. The variety of batteries and the increasing use of electronics and lightweight design have led to an increased total use of metals, especially in BEVs. At the same time, the amount of plastics in a car is increasing, while the amount of steel is decreasing. The fraction of ASR contributes between 15 and 25 wt % of an ELV and is expected to grow in the future. ASR poses the most significant challenge when considering the recycling of a vehicle.^59,60^ Due to its inherent characteristics, incineration or pyrolysis results in a noncombustible residue comprising glass, rock, sand, and residual metals, including rare earth elements. The increasing demand for these metals necessitates the development of effective recycling strategies, with little work done on metal recovery from ASR.^66,67^ In aiming at a circular economy, these tasks need to be addressed. For example, to reach circular plastics on a global scale, their production and recycling methods need to be changed fundamentally.^68^ Recent advances in mechanical recycling of plastic waste alone will not suffice.^69^ Research on new circular plastics or adhesives that allow for debonding on demand will not succeed on their own.^70–74^ Circular plastics are only one step toward a recyclable car. Multiple scientific fields must work together toward this goal. We encourage material chemists to take off the blinders and underpin claims of newly developed sustainable materials with appropriate life cycle assessment. This is especially cumbersome at a low technology readiness level due to the lack of standardized data,^75^ yet recent strategies provide the tools and invite for collaboration.^76–79^

## Carbon Neutral Materials from Renewables

In addition to recycling strategies, such as closing the technical cycle, renewable resources can be utilized for biodegradable materials to close the biological cycle (Figure 2). Biomass, an important renewable resource and highly functionalized feedstock, can contribute to automotive plastics by giving rise to platform chemicals that can be converted into building blocks for polymer production.^80^ Biobased plastics utilize carbon that has been absorbed through photosynthesis, thus degradation releases carbon previously incorporated from the atmosphere to create a closed-loop carbon cycle.^81^ In modern vehicles, six primary polymers, including PP, polyurethane, polyamide (PA), polyethylene, acrylonitrile butadiene styrene/styrene acrylonitrile and polyethylene terephthalate (PET), constitute over 80% of total plastic content and are predominantly utilized in high-functionality components such as bumpers, dashboards and seats.^82^ Substituting these polymers with renewably sourced plastics that foster closed-loop recycling, is a major lever toward a recyclable car.

A collaboration of Ford and Coca-Cola on sugar cane-based PET for seat fabrics demonstrates the potential of biomass to displace fossil plastics.^83^ Partially biobased PA (70% from castor plants) has been used by Mercedes-Benz to reduce weight and emissions.^83^ Other plastics such as polylactic acid (PLA) or polyhydroxyalkanoates derived from plant or microbial sources can be used to make vehicle interior components, panels and body parts.^84^ In 2021, only about 2% of plastics in Europe were made from biomass but the share of biobased plastics will continue to grow and bolster Europe’s transition toward a circular economy.^85^ Components of the car’s interior and exterior, particularly tires, present a significant challenge due to the unavoidable abrasion of tire material and the subsequent runoff into the environment. The tire material itself is not biodegradable, and additives such as antiozonants impact the ecosystem.^86–88^ The tire industry is investing in ways to make tires more sustainable by replacing these additives with biobased and degradable alternatives.^89,90^

Future developments in the automotive sector have to be based on closed-loop thinking.

The potential of renewable feedstocks extends well beyond plastics. Biobased lubricants,^91–93^ adhesives,^94^ coatings,^95^ additives,^96^ and sealants^97^ are being developed (Figure 3). These products harness the inherent chemical diversity of biomass to offer performance characteristics comparable to, or even surpassing, their fossil-based counterparts. More importantly, substituting nonrenewable energy sources (e.g., natural gas and petroleum) with renewable alternatives (e.g., wind, solar, biomass, and hydropower) during the material production phase can substantially lower the associated process carbon footprint, thereby supporting a more sustainable material supply chain and contributing to the reduction of total life cycle greenhouse gas emissions within the automotive sector.^98–104^

**Figure 3:**
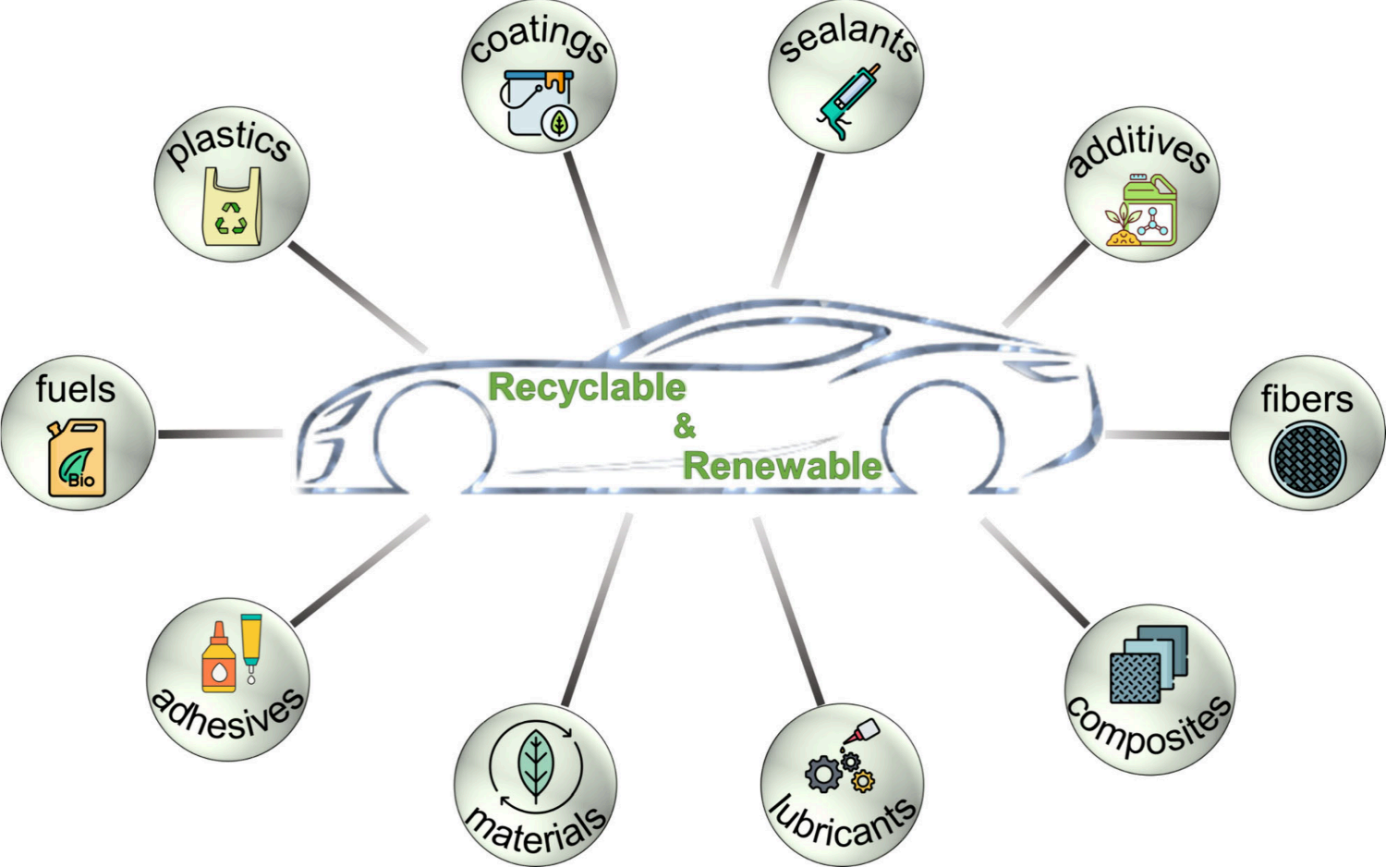
Biobased chemicals and materials pave the way toward a fully recyclable car.

Despite the promise of renewable feedstocks, multiple technical and economic hurdles have to be overcome prior to their widespread adoption in the automotive industry. A key challenge involves ensuring that biobased materials meet the demanding durability, safety, and performance criteria required for vehicles operating under extreme conditions, including temperature fluctuations, UV exposure, and mechanical stress. Nevertheless, advances in biobased polymer engineering, the reinforcement of natural fibers, and the development of hybrid composite formulations are enhancing resilience and longevity, making these materials increasingly viable for automotive applications.^105^ Early biobased polymers like PLA often were too brittle and not sufficiently heat resistant, but innovations in hybrid composites, like lignin-reinforced PLA and natural fiber-enhanced polyurethanes, have achieved performance parity with traditional plastics, enabling their use in critical components like engine covers and seat foams.^106^

For us, recycling should mean cradle-to-cradle design since by truly closing the loop, a continuous, sustainable, and truly circular economy is within reach.

A desire to reduce CO_2_ emissions and fuel consumption has led the automotive industry to develop lighter vehicles. A 10% weight reduction can lower fuel usage by 6–8% and emissions by 5–6%. Lighter cars need less energy, boosting fuel efficiency in ICEVs and extending the range of BEVs, thereby reducing environmental impact. Replacing steel with plastics and composites, poses additional challenges for recycling efforts. Future cars need to be conceived with the goal of creating a recyclable ELV. Closed-loop recycling in automotive industry would be significantly easier when monomaterials derived from renewable feedstocks are used that combine a unified chemical composition with multifunctional performance.^107^ Materials can be combined when separation strategies exist either directly or by breaking them down for separation at the molecular level. The demand for new materials designed to be recycled is on the rise.

## Accelerating Material Research by Data-Driven Chemistry

Novel materials with properties tailored for sustainability represent a fundamental step in designing fully recyclable vehicles. The materials discovery process involves four main stages: defining a research question, collecting relevant data, formulating hypotheses, and conducting experimental validation. AI-driven predictive models facilitate the identification of new materials with desirable recycling properties, accelerating their development and reducing the reliance on resource-intensive trial-and-error methods. Deep learning algorithms have been employed to model chemical structures, predict molecular properties, and optimize reaction pathways, significantly shortening the time required for material innovation.^108–113^

The graph networks for materials exploration (GNoME) developed by DeepMind, predicts the electron density distributions of molecules and enables researchers to calculate molecular properties such as dipole moments and reaction mechanisms with high precision and speed. The technique bypasses the computational bottlenecks of traditional quantum mechanical simulations, paving the way for broader accessibility to accurate molecular data to enhancing our understanding of chemical systems.^114^ This approach leverages a combination of different algorithms trained at scale on the available data to explore and filter candidate structures. GNoME led to a substantial increase in the identification of stable inorganic crystal structures compared to previous in-silico approaches.^115^ The predictions were validated by autonomous robotic experiments, yielding an impressive 71% success rate. The newly discovered materials data is publicly available through the Materials Project Database, offering researchers the opportunity to identify materials with desired properties for various applications.

Switch to renewable feedstocks and renewable energy sources to reduce automotive emissions from the ground up.

Generative models offer an even more powerful approach. Several (deep) generative models including, Variational Autoencoders, Adversarial Autoencoders, Objective-Reinforced Generative Adversarial Networks, Character-level Recurrent Neural Network), REINVENT, and GraphINVENT helped to design denovo (retro)­synthesis routes^116^ for complex materials and to predict novel molecular structures that satisfy predefined property requirements.^117–119^ Recent advances exemplify the transformative capability by integrating Generative Adversarial Networks with polymer self-consistent field theory to discover new block polymer phases.^120^

To further reduce the time required for material development and testing, generative AI can be integrated with high-throughput experimental validation. The Materials Genome Initiative^121,122^ focuses on reducing the ″discovery-to-deployment″ timeline for new materials,^123^ aiming to cut development time from 20 to less than ten years.

Despite its transformative potential, harnessing the full potential of machine learning (ML) and generative AI in the chemical domain remains extremely challenging (Figure 4). Data availability and quality remain principal constraints, as robust models require comprehensive, high-quality data sets that capture the diversity of relevant chemical systems. Inconsistent labeling and incomplete records in materials data sets often hinder effective ML training, underscoring the need for standardized data collection practices.^110^

**Figure 4:**
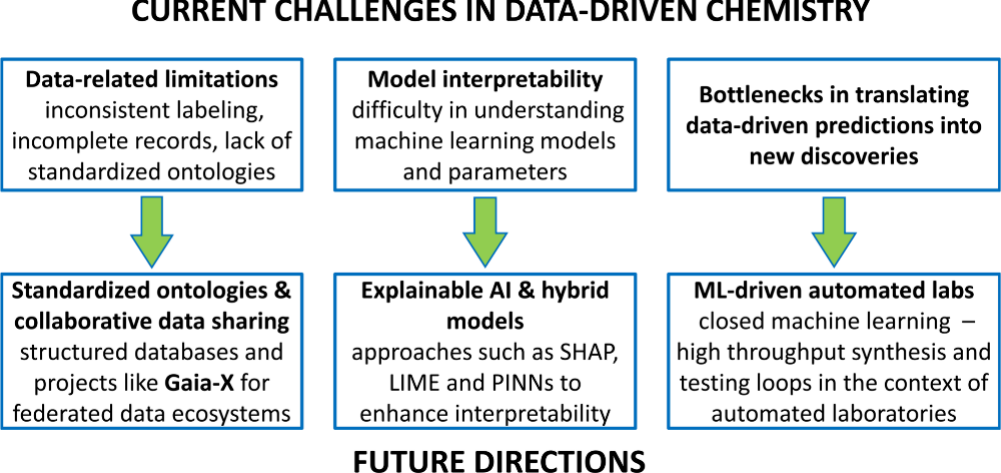
Current challenges in data-driven chemistry and ways to address them. (SHAP: SHapley Additive exPlanations; LIME: Local Interpretable Model-agnostic Explanations; PINN: Physics-Informed Neural Networks).

We encourage material chemists to take off the blinders and underpin claims of newly developed sustainable materials with appropriate life cycle assessment.

Despite breakthroughs like AlphaFold for protein folding, the adoption of AI in chemistry remains limited: “*For chemists, the AI revolution has yet to happen*”^124^ as current efforts in AI primarily focus on narrow applications rather than addressing broader, more complex chemical challenges. Integration of diverse data sets, standardized reporting, and interdisciplinary collaboration are required to fully unlock AI’s transformative potential in chemistry.

Collaborative initiatives involving academic institutions, industry stakeholders, and government agencies are imperative to foster data sharing. Programs like the Materials Genome Initiative^121,122^ have successfully demonstrated the value of integrating computational tools with experimental databases, providing a framework to inspire further efforts in key productive sectors such as the automotive industry.

Collaborative projects such as Gaia-X,^125^ aim to create secure, interoperable data ecosystems in Europe, supporting data sovereignty and transparency. Integrating proprietary and public data sets is crucial for material science. Gaia-X could unify data on polymer recyclability and alloy performance, enabling seamless sharing among researchers, manufacturers and policymakers to enhance material discovery for end-of-life recovery. Its federated approach keeps sensitive data secure while supporting sustainable automotive innovation. Using Gaia-X and the automotive network Catena-X, researchers can aggregate diverse data sources, forming a platform for high-quality data sets in sustainable automotive design.^125,126^

Ontological databases support advanced machine learning algorithms by providing well-structured data. Graph-based ML models capture relational properties and can integrate with these databases to improve prediction interpretability. This synergy between data architecture and algorithms can accelerate the discovery of new materials with optimized performance. Recent advances have shown that AI can be effectively integrated into traditional lab environments to enable generalizable chemical synthesis. A modular, cloud-based workflow for organic solid-state laser discovery, for instance, used GNN-informed Bayesian optimization to explore over 150,000 candidates, achieving a 74% success rate.^127^ Similarly, a closed-loop ML system for heteroaryl Suzuki–Miyaura couplings identified reaction conditions that doubled average yields over established benchmarks.^128^ These studies demonstrate that AI-guided strategies, compatible with standard lab infrastructure, can transform chemical discovery by efficiently navigating vast experimental spaces. However, their success critically depends on standardized data formats and access to high-quality, diverse data sets to train reliable models.

Looking ahead, the synergistic application of ML and generative AI will play a central role in material science for sustainable automotive design. The maturation of these technologies and their incorporation into autonomous laboratories and high-throughput experimental platforms hold the potential to revolutionize materials discovery and process optimization. By harnessing the convergence of computational innovation and chemical expertise, the vision of a fully recyclable car is poised to become a tangible reality, marking a significant milestone in sustainable engineering. As these efforts progress, the integration of interdisciplinary research, policy frameworks, and international collaboration will be vital in realizing the full potential of these transformative technologies.

Success critically depends on standardized data formats and access to high-quality, diverse datasets to train reliable models.

## Conclusion

Fully recyclable cars will reduce environmental impact and improve resource efficiency in the automotive industry. Currently used materials need to be evaluated and new materials, designed for recycling and based on nonfossil resources will be developed. Creating a recyclable car can be considered “Chemistry’s Moonshot”, serving as an overarching goal to guide scientific efforts and align different areas of chemical research. Chemistry, as a foundational discipline, is the base for the automotive industry as well as virtually all economic sectors. The recyclable car, based on renewable, recyclable materials will shape future materials development. At the same time, the field will have to transform how chemical research is performed. Growing piles of plastic waste and climate change are not patiently waiting for us to develop solutions and chemists need to find ways to accelerate their research. Artificial Intelligence can fundamentally change our approach to many chemical problems as some pioneering studies cited above illustrate. The quality of data chemists generate will determine the impact AI can make in reshaping materials in the automotive industry and beyond.

## Supplementary Material



## Notes

Transparent Peer Review report available (PDF)
