# Supplementary material for: Chemistries Moonshot: An Entirely Recyclable Car
Source: ACS Cent Sci. 2025 Jul 2;11(7):1052–61. doi: 10.1021/acscentsci.5c00589 (PMC12291114; doi:10.1021/acscentsci.5c00589)
Supplement: Supplementary file 1 [file oc5c00589_si_001.pdf]

oc-2025-00589f.R1

Name: Peer Review Information for "Chemistries Moonshot: An Entirely Recyclable Car"

First Round of Reviewer Comments

Reviewer: 1

Comments to the Author

In this Outlook, Seeberger, Tyrikos-Ergas, and colleagues present an ambitious and thought-provoking vision for an “entirely recyclable car,” emphasizing the critical need for collaboration between the automotive and chemical industries. The authors also highlight the growing role of predictive models and generative AI in enabling the discovery of sustainable materials, a timely and promising area of research. I commend the authors for tackling a high-risk, forward-looking concept that aligns well with the broader sustainability goals of the scientific and industrial communities. The discussion around generative AI and high-throughput experimentation is particularly compelling, and Figure 4 provides a well-articulated set of actionable ideas that underscore the potential of these technologies to accelerate progress toward recyclable automotive systems. However, I found the Outlook would benefit from greater specificity, particularly in outlining realistic pathways for transitioning from our contemporary reliance on fossil fuels to more sustainable materials. Some of the sections – especially those discussing the development of new renewable materials – were in my view broad and speculative, lacking the technical detail that would help guide researchers or stakeholders in the field. In contrast, the sections focused on AI were more concrete and effective in conveying next steps. Additionally, an important omission is the role of renewable energy as a foundational element for enabling the sustainable production of bio-based materials. The complex interdependencies among energy, water, and feedstock sourcing – especially in the context of the water-energy nexus – deserve mention. Addressing this would provide a more holistic view of the systemic challenges involved.

This Outlook has strong potential to resonate with the multidisciplinary readership of ACS Central Science. However, to better inform and inspire the scientific community, I think that

the manuscript requires major revisions to improve technical specificity, interdisciplinary framing, and practical relevance. I have included additional comments below to assist in strengthening the submission.

Major Comments:

1. ToC Graphic Transparency and Clarity. Upon viewing the Table of Contents (ToC) graphic, I wondered whether it was generated using an AI platform, based on stylized text artifacts such as “Circular Learning” and what appears to be “Sercala… Ecoron” (the latter being cut off or nonsensical). Given the Outlook’s emphasis on AI, this is not necessarily a drawback, but transparency would be helpful. If the image was AI-generated, I recommend disclosing this in the figure caption or supporting information. If not, the authors should consider clarifying or correcting the text to ensure it is legible and purposeful. Visually, the graphic is engaging and fits the theme well.

2. Figure Resolution and Legibility. Several figures in the main text, particularly Figures 1 and 4, are low in resolution with small, difficult-to-read text. I recommend the authors improve the graphical quality and enhance legibility to ensure accessibility to a broad readership.

3. Figure 1 – Emissions Breakdown and Context. In Figure 1 (right), the bar chart would be more informative if it presented absolute rather than normalized emission values for ICEVs and BEVs. This would provide better context for understanding where emissions originate and where mitigation efforts might be most impactful. Additionally, expanding the breakdown of emissions sources within material production would help readers grasp the specific contributions of various components.

4. Disciplinary Framing of the Recyclable Car Vision. I encourage the authors to reconsider the framing of the recyclable car as a “single, unifying project in material chemistry.” While materials chemistry will play a central role, such a grand challenge is inherently interdisciplinary, requiring input from systems engineering, industrial ecology, sustainability science, and beyond. Suggesting otherwise risks oversimplifying the complexity of the effort. Similarly, the statement that chemistry lacks a unifying initiative due to its diversity – especially when contrasted with projects like the Apollo Program or Human Genome Project – is difficult to substantiate and may unintentionally diminish the interdisciplinary nature of those efforts. If the authors’ intent is to highlight the potential for chemistry to unify around a major sustainability objective, I recommend they reframe this aspiration in more inclusive and evidence-based terms.

5. Terminology Around Circularity. The distinction between “biological” and “technical” cycles is helpful and well explained. To further strengthen this section, the authors might consider referencing commonly used terms such as “circular economy” or “circular plastics,” which distinguish closed-loop recycling from forms of up- or down-cycling. This would connect their discussion to broader academic and policy conversations around sustainability.

6. Broader Disciplinary Engagement with Recycling Technologies. The Outlook emphasizes the role of chemical recycling, but recent arguments suggest that advances in separations and mechanical recycling – often driven by engineering disciplines – may be more immediately scalable. While the centrality of chemistry remains clear, expanding the discussion to reflect contributions from chemical, mechanical, and systems engineering would present a more comprehensive and realistic view of the innovation landscape. Recognizing these synergies would not diminish the manuscript’s fit for ACS Central Science, but rather enhance its appeal to a multidisciplinary audience.

7. Energy Demands of Bio-Based Materials. While the Outlook rightly champions bio-based feedstocks, it omits an important consideration: the energy inputs required to produce these materials are often derived from fossil fuels. This is a nontrivial issue that complicates the environmental benefits of renewable materials. Acknowledging the role of renewable energy in enabling a truly sustainable feedstock supply chain would provide a more balanced view. Additionally, if space permits, including even brief quantitative comparisons of energy use or emissions (e.g., in relation to the water-energy nexus) could strengthen this section considerably.

8. Integration of AI in Traditional Labs. The section on artificial intelligence could be made even more impactful by providing concrete suggestions for how traditional chemistry labs might begin integrating AI tools. Including a brief discussion of common pitfalls would be particularly helpful for readers unfamiliar with these tools.

Author's Response to Peer Review Comments:

Deputy Editor

ACS Central Science

June 19th, 2025

Manuscript ID: oc-2025-00589f

Original Submission Date: 10-Apr-2025

Title: "Chemistries Moonshot: An Entirely Recyclable Car"

Author(s): Schoemaker, Robin; Sun, Chunning; Chiarugi, Davide; Tyrikos-Ergas, Theodore; Seeberger, Peter.

Dear Editor,

Thank you for taking the time to evaluate our work and forwarding the useful comments of the referee. We found the comments to be very helpful, and we have addressed all the latter in a revised manuscript. All changes in the revised manuscript are clearly marked. Below is a detailed point-by-point response to reviewer comments.

General comments

Comment 1: “page 3, delete "this" - Thinking of recycling as a loop of matter _this_ is actually…”

Response: Corrected.

Comment 2: “Consider reducing the use of acronyms; in some places these are cumbersome and decrease readability by a general reader.”

Response: We significantly reduced the use of acronyms, particularly eliminating those that were redundant or used only a single time.

Comment 3: “Copyright permissions are required for any graphic that is reproduced or adapted from a source that is not published by the American Chemical Society. See, for example, Figures 1 and 2. Please make sure that all graphics reproduced or adapted from another journal have the proper permission forms and credit lines in your revision.”

Response: Thank you for your note. We are aware of the ACS policy regarding the use of graphics from other sources. However, we would like to clarify that Figures 1 and 2, as well as all other figures in the manuscript, were created entirely by us and are not reproduced or adapted from any previously published material.

Reviewer:

Comment 1:

“ToC Graphic Transparency and Clarity. Upon viewing the Table of Contents (ToC) graphic, I wondered whether it was generated using an AI platform, based on stylized text artifacts such as “Circular Learning” and what appears to be “Sercala… Ecoron” (the latter being cut off or nonsensical). Given the Outlook’s emphasis on AI, this is not necessarily a drawback, but transparency would be helpful. If the image was AI-generated, I recommend disclosing this in the figure caption or supporting information. If not, the authors should consider clarifying or correcting the text to ensure it is legible and purposeful. Visually, the graphic is engaging and fits the theme well.”

Response: We have added a description to the TOC caption that it was generated with ChatGPT v4 and further edited using Adobe Photoshop v25.0. We also removed the nonsensical text that was in the background.

Comment 2:

“Figure Resolution and Legibility. Several figures in the main text, particularly Figures 1 and 4, are low in resolution with small, difficult-to-read text. I recommend the authors improve the graphical quality and enhance legibility to ensure accessibility to a broad readership.”

Response: We have increased the text size and improved the image resolution.

Comment 3:

“Figure 1 – Emissions Breakdown and Context. In Figure 1 (right), the bar chart would be more informative if it presented absolute rather than normalized emission values for ICEVs and BEVs. This would provide better context for understanding where emissions originate and where mitigation efforts might be most impactful. Additionally, expanding the breakdown of emissions sources within material production would help readers grasp the specific contributions of various components.”

Response: We appreciate the suggestions; however, absolute emission values for ICEVs and BEVs are not readily available, as some data are based on calculations. Emissions from material production vary across industrial sectors. We have included relevant literature as reference for the readers.

Comment 4:

"Disciplinary Framing of the Recyclable Car Vision. I encourage the authors to reconsider the framing of the recyclable car as a "single, unifying project in material chemistry." While materials chemistry will play a central role, such a grand challenge is inherently interdisciplinary, requiring input from systems engineering, industrial ecology, sustainability science, and beyond. Suggesting otherwise risks oversimplifying the complexity of the effort. Similarly, the statement that chemistry lacks a unifying initiative due to its diversity – especially when contrasted with projects like the Apollo Program or Human Genome Project – is difficult to substantiate and may unintentionally diminish the interdisciplinary nature of those efforts. If the authors' intent is to highlight the potential for chemistry to unify around a major sustainability objective, I recommend they reframe this aspiration in more inclusive and evidence-based terms."

Response: We reframed chemistry's moonshot in this context and elaborated its interdisciplinary character as well as noting the effect of the other moonshots on a wide field of scientific disciplines. Moreover, we rewrote the introduction, inviting other disciplines to join this journey to the moon. We especially would like to thank the reviewer for noting the importance of systems engineering and the like, which is a crucial part starting from early material research to the full life cycle of materials

We added the following text:

"While chemistry will be a major part of this grand challenge, it goes beyond that due to its inherent interdisciplinary nature. In addition to new materials, a new way of thinking about these materials is necessary. This new way of thinking should start with research including assumptions on life cycles. Therefore, material chemists need input from systems engineering, industrial ecology and sustainability science, especially when thinking of new developments in the field of data sciences and AI.15 The goal is to holistically develop materials from cradle to cradle instead of synthesizing them to perform before discarding them.16 Materials chemistry is inviting all scientific fields in question from automotive, environmental or systems engineering to data science, recycling and waste management on a journey to the moon."

Comment 5:

“Terminology Around Circularity. The distinction between “biological” and “technical” cycles is helpful and well explained. To further strengthen this section, the authors might consider referencing commonly used terms such as “circular economy” or “circular plastics,” which distinguish closed-loop recycling from forms of up- or down-cycling. This would connect their discussion to broader academic and policy conversations around sustainability.

Response: We thank the reviewer for this valuable recommendation, which we have gladly implemented.

Comment 6:

“Broader Disciplinary Engagement with Recycling Technologies. The Outlook emphasizes the role of chemical recycling, but recent arguments suggest that advances in separations and mechanical recycling – often driven by engineering disciplines – may be more immediately scalable. While the centrality of chemistry remains clear, expanding the discussion to reflect contributions from chemical, mechanical, and systems engineering would present a more comprehensive and realistic view of the innovation landscape. Recognizing these synergies would not diminish the manuscript’s fit for ACS Central Science, but rather enhance its appeal to a multidisciplinary audience.”

Response: We agree with the reviewer and widened the scope by pointing out mechanical and metallurgical separation and recycling with the focus still remaining on chemistry closely connected to systems engineering.

We added the following text:

“In aiming at a circular economy, these tasks need to be addressed. For example, to reach circular plastics on a global scale, their production and recycling methods need to be changed fundamentally.68 Recent advances in mechanical recycling of plastic waste alone will not suffice.69 Research on new circular plastics or adhesives that allow for debonding on demand will not succeed on their own.70-74 Circular plastics are only one step towards a recyclable car. Multiple scientific fields must work together towards this goal. We encourage material chemists to take off the blinders and underpin claims of newly developed sustainable materials with appropriate life cycle assessment. This is especially cumbersome at a low technology readiness level due to the lack of standardized data,75 yet recent strategies provide the tools and invite for collaboration.76-79”

Comment 7:

“Energy Demands of Bio-Based Materials. While the Outlook rightly champions bio-based feedstocks, it omits an important consideration: the energy inputs required to produce

these materials are often derived from fossil fuels. This is a nontrivial issue that complicates the environmental benefits of renewable materials. Acknowledging the role of renewable energy in enabling a truly sustainable feedstock supply chain would provide a more balanced view. Additionally, if space permits, including even brief quantitative comparisons of energy use or emissions. (e.g., in relation to the water-energy nexus) could strengthen this section considerably."

Response: Renewable energy input is important for sustainable materials production. A description has been added to the main text.

"More importantly, substituting non-renewable energy sources (e.g., natural gas and petroleum) with renewable alternatives (e.g., wind, solar, biomass, and hydropower) during the material production phase can substantially lower the associated process carbon footprint, thereby supporting a more sustainable material supply chain and contributing to the reduction of total life cycle greenhouse gas emissions within the automotive sector.98-104"

Due to space limitations, we cannot provide comparisons among different energy inputs, but relevant literature has been included in the Outlook for readers' reference.

Comment 8:

"Integration of AI in Traditional Labs. The section on artificial intelligence could be made even more impactful by providing concrete suggestions for how traditional chemistry labs might begin integrating AI tools. Including a brief discussion of common pitfalls would be particularly helpful for readers unfamiliar with these tools."

Response:

We appreciate the reviewer's insightful comment regarding the integration of AI in traditional chemistry labs. In response, we have added examples from recent literature that illustrate how AI-driven workflows have been successfully implemented in standard lab environments. We also now explicitly mention the critical pitfall of ensuring access to reliable, high-quality data for effective model development.

We added the following text:

"Recent advances have shown that AI can be effectively integrated into traditional lab environments to enable generalizable chemical synthesis. A modular, cloud-based workflow for organic solid-state laser discovery, for instance, used GNN-informed Bayesian optimization to explore over 150,000 candidates, achieving a 74% success rate.127 Similarly, a closed-loop ML system for heteroaryl Suzuki–Miyaura couplings identified reaction conditions that doubled average yields over established benchmarks.128 These

studies demonstrate that AI-guided strategies, compatible with standard lab infrastructure, can transform chemical discovery by efficiently navigating vast experimental spaces. However, their success critically depends on standardized data formats and access to high-quality, diverse datasets to train reliable models."

Thank you for the opportunity to submit a revised manuscript that has been greatly improved by the constructive comments of the reviewer and the editorial team. We hope that you will now find it suitable for publication in ACS Central Science.

Sincerely yours,

Prof. Dr. Peter H. Seeberger
